# Prognóstico e Características Associadas a Trombose de Prótese Valvar: Insights de um Estudo Brasileiro

**DOI:** 10.36660/abc.20220739

**Published:** 2022-11-01

**Authors:** Giovanni Possamai Dutra, Bruno Ferraz de Oliveira Gomes

**Affiliations:** 1 Hospital Barra D’Or Rio de Janeiro RJ Brasil Hospital Barra D’Or, Rio de Janeiro, RJ – Brasil; 2 Universidade Federal do Rio de Janeiro Rio de Janeiro RJ Brasil Universidade Federal do Rio de Janeiro, Rio de Janeiro, RJ – Brasil

**Keywords:** Trombose, Prótese Valvar, Prognóstico

Desde a década de 60, a doença valvar cardíaca passou por importantes mudanças em relação a sua estratégia terapêutica. O início do emprego das cirurgias de substituição valvar utilizando próteses modificou o prognóstico de pacientes com doença valvar em todo o mundo. Anualmente, são implantadas mais de 280 mil próteses valvares.^1^

A incidência de doença valvar de etiologia degenerativa, tem aumentado nos países industrializados enquanto, infelizmente, a doença cardíaca reumática ainda é observada com frequência em muitas partes do mundo, sendo a etiologia mais prevalente de valvopatia no Brasil. A prótese valvar mecânica é a mais indicada para pacientes mais jovens, muitas vezes acometidos pela doença reumática, explicando sua relevância em nosso país.^2–4^

A trombose de prótese valvar é um evento incomum sendo mais frequente em próteses mecânicas, principalmente em posição mitral.^5^ Este evento é uma das complicações mais graves do pós-operatório de troca valvar, com incidência anual variável entre 0,5 e 6%^6^ e possui alta mortalidade, que, em alguns estudos, pode ultrapassar 30%.^7^

O estudo “Aspectos Clínicos e de Sobrevida de Pacientes pós Implante de Valva Mecânica, com Ênfase em Trombose de Prótese Valvar” trouxe uma importante visão sobre esse tema.^8^ Trata-se de uma grande coorte retrospectiva onde foram identificados 473 implantes de próteses mecânicas de 2011 a 2017. Por ser um estudo conduzido no Brasil, a doença reumática foi a principal causa de troca valvar, justificando o perfil de idade mais jovem desta população. Em contrapartida, a troca valvar aórtica foi mais prevalente (49,9%) seguida da troca mitro-aórtica (30,2%) e mitral (19,9%). Os autores justificaram esses achados baseados em duas hipóteses: (1) preferência de biopróteses em mulheres jovens e/ou (2) possibilidade de intervenção por valvuloplastia percutânea na valvopatia mitral.

A mortalidade geral observada neste estudo foi um pouco mais elevada em comparação a outros estudos, apesar de existir uma grande heterogeneidade nas populações estudadas. De qualquer forma, a mortalidade geral foi menor que a observada nacionalmente. Alguns estudos já demonstraram que o uso de próteses mecânicas confere maior sobrevida em populações mais jovens,^9^ com mortalidade de 26,4% em 15 anos. O estudo de Tagliari encontrou uma mortalidade de 16% no seguimento médio de 4,4 anos. Destaca-se que a classe funcional após a cirurgia e insuficiência renal crônica foram as principais variáveis associadas a mortalidade.

A trombose de prótese valvar foi um evento raro, semelhante aos dados disponíveis na literatura. Além disso, observamos que é um evento geralmente tardio, com tempo médio de ocorrência de 39 meses. A varfarina é o anticoagulante de eleição no paciente portador de prótese mecânica. No entanto, seu perfil farmacológico que promove flutuações no nível terapêutico, podem expor o paciente à um maior risco de trombose. Neste estudo, o INR dos pacientes que apresentaram trombose não apresentou diferença aos que não apresentaram trombose, demonstrando que outros fatores podem estar envolvidos. A formação do *pannus*, fator pró-trombótico conhecido, associou-se à maior ocorrência de trombose de prótese. Por fim, o tabagismo, outro fator sabidamente pró-trombótico, também manifestou associação. Dessa forma, a identificação de fatores que aumentam o risco de trombose deve ser avaliada nesses pacientes rotineiramente.

O sangramento é uma complicação temida em pacientes portadores de prótese valvar. Sabemos que o risco de sangramento é maior nessa população, comparada aos portadores de biopróteses.^10^ No estudo de Tagliari, esta complicação aconteceu em 23 doentes (4,86%), sendo que todos demandaram internação hospitalar. O sangramento foi responsável pela morte de 2 doentes. Em estudo de Labaf et al, idade e sangramento prévio foram importantes preditores de sangramento.^11^ Em pacientes com prótese mecânica mitral, insuficiência renal também foi um preditor importante.

O estudo de Tagliari é, portanto, um interessante registro sobre a doença valvar em nossa população. Sobretudo por mostrar a jovem população acometida por doença reumática com alta frequência de realização de troca valvar mecânica e suas complicações. A mortalidade da população estudada é elevada, em concordância com as variações observadas na literatura mundial. O status funcional e a presença de insuficiência renal crônica foram associados a maior mortalidade. O tabagismo e a presença de *pannus* foram destacados como fatores a serem atentamente observados neste grupo de pacientes considerando a hipótese levantada por este artigo de sua relação com trombose de prótese. Tais achados reforçam a importância da indicação correta de troca valvar e seguimento adequado desta população.
