# Supplementary material for: Prognóstico e Características Associadas a Trombose de Prótese Valvar: Insights de um Estudo Brasileiro
Source: Arq Bras Cardiol. 2022 Nov 1;119(5):745–6. [Article in Portuguese] doi: 10.36660/abc.20220739 (PMC9750219; doi:10.36660/abc.20220739)
Supplement: Translation

# Translation: Prognosis and Characteristics Associated with Prosthetic Valve Thrombosis: Insights from a Brazilian Study

**Authors:** Giovanni Possamai Dutra, Bruno Ferraz de Oliveira Gomes

**Affiliations:** 1 Rio de Janeiro RJ Brazil Hospital Barra D’Or, Rio de Janeiro, RJ – Brazil; 2 Rio de Janeiro RJ Brazil Universidade Federal do Rio de Janeiro, Rio de Janeiro, RJ – Brazil

**Keywords:** Thrombosis, Prosthetic Valve, Prognosis

Since the 1960s, heart valve disease has undergone critical changes in its therapeutic strategy. The beginning of valve replacement surgeries using prostheses changed the prognosis of patients with valve disease worldwide. Annually, more than 280 thousand valve prostheses are implanted.^1^

The incidence of valvular disease of degenerative etiology has increased in industrialized countries while, unfortunately, rheumatic heart disease is still often observed in many parts of the world, being the most prevalent etiology of valvular heart disease in Brazil. The mechanical valve prosthesis is the most indicated for younger patients, often affected by the rheumatic disease, which explains its relevance in our country.^2–4^

A prosthetic valve thrombosis is an uncommon event that is more frequent in mechanical prostheses, especially in the mitral position.^5^ This event is one of the most serious complications in the postoperative period of valve replacement, with an annual incidence that varies between 0.5 and 6%^6^ and a high mortality rate, which, in some studies, can exceed 30%.^7^

The study “Clinical Features and Survival Analysis of Patients after Mechanical Heart Valve Replacement, with an Emphasis on Prosthetic Valve Thrombosis” brought important insight into this topic.^8^ This is a large retrospective cohort in which 473 mechanical prosthesis surgeries were identified from 2011 to 2017. To a study conducted in Brazil, the rheumatic disease was the main cause of valve replacement, justifying the younger age profile of this population. In contrast, aortic valve replacement was more prevalent (49.9%), followed by mitral-aortic valve replacement (30.2%) and mitral valve replacement (19.9%). The authors justified these findings based on two hypotheses: (1) preference for bioprosthesis in young women and/or (2) the possibility of intervention by percutaneous valvuloplasty in mitral valve disease.

The overall mortality observed in this study was slightly higher than in other studies, despite the great heterogeneity in the studied populations. In any case, overall mortality was lower than that observed nationally. Some studies have already shown that mechanical prostheses lead to greater survival in younger populations,^9^ with a mortality rate of 26.4% in 15 years. Tagliari’s study found a mortality of 16% at a mean follow-up of 4.4 years. Notably, the functional class after surgery and chronic renal failure were the main variables associated with mortality.

Prosthetic valve thrombosis was a rare event, similar to the data available in the literature. In addition, we observed that it is a generally late event, with a mean time of occurrence of 39 months. Warfarin is the anticoagulant of choice in patients with mechanical prostheses. However, its pharmacological profile, which promotes fluctuations in the therapeutic level, may expose the patient to a greater risk of thrombosis. In this study, the RNI of patients with thrombosis showed no difference from those who did not have thrombosis, showing that other factors may be involved. The formation of pannus, a known prothrombotic factor, was associated with a higher occurrence of prosthesis thrombosis. Finally, smoking, another known prothrombotic factor, was also associated. Thus, these patients should routinely evaluate identifying factors that increase the risk of thrombosis.

Bleeding is a feared complication in patients with prosthetic valves. We know that the risk of bleeding is higher in this population compared to patients with bioprosthesis.^10^ In Tagliari’s study, this complication occurred in 23 patients (4.86%), all of whom required hospitalization. Bleeding was responsible for the death of 2 patients. In a study by Labaf et al., age and previous bleeding were important predictors of bleeding.^11^ Chronic kideny failure was also an important predictor in patients with mechanical mitral valve prosthesis.

Tagliari’s study is an interesting record of valve disease in our population. Especially for showing the young population affected by the rheumatic disease with a high frequency of mechanical valve replacement and its complications. The mortality of the studied population was high, in agreement with the variations observed in the world literature. Functional status and chronic renal failure were associated with higher mortality. Smoking and pannus were highlighted as factors to be carefully observed in this group of patients, considering the hypothesis raised by this article of its relationship with prosthesis thrombosis. Such findings reinforce the importance of the correct indication of valve replacement and adequate follow-up of this population.
